# Gene Expression, Biochemical Characterization of a sn-1, 3 Extracellular Lipase From *Aspergillus niger* GZUF36 and Its Model-Structure Analysis

**DOI:** 10.3389/fmicb.2021.633489

**Published:** 2021-03-12

**Authors:** Shuqi Xing, Ruonan Zhu, Kai Cheng, Yangyang Cai, Yuedan Hu, Cuiqin Li, Xuefeng Zeng, Qiujin Zhu, Laping He

**Affiliations:** ^1^Key Laboratory of Agricultural and Animal Products Store and Processing of Guizhou Province, Guizhou University, Guiyang, China; ^2^College of Liquor and Food Engineering, Guizhou University, Guiyang, China; ^3^School of Chemistry and Chemical Engineering, Guizhou University, Guiyang, China; ^4^Guizhou Province Key Laboratory of Fermentation Engineering and Biopharmacy, Guizhou University, Guiyang, China

**Keywords:** *Aspergillus niger* lipase, cloning and expression, biochemical characterization, homology modeling, molecular docking, SAXS analysis

## Abstract

In this study, a sn-1, 3 extracellular lipases from *Aspergillus niger* GZUF36 (PEXANL1) was expressed in *Pichia pastoris*, characterized, and the predicted structural model was analyzed. The optimized culture conditions of *P. pastoris* showed that the highest lipase activity of 66.5 ± 1.4 U/mL (*P* < 0.05) could be attained with 1% methanol and 96 h induction time. The purified PEXANL1 exhibited the highest activity at pH 4.0 and 40°C temperature, and its original activity remained unaltered in the majority of the organic solvents (20% v/v concentration). Triton X-100, Tween 20, Tween 80, and SDS at a concentration of 0.01% (w/v) enhanced, and all the metal ions tested inhibited activity of purified PEXANL. The results of ultrasound-assisted PEXANL1 catalyzed synthesis of 1,3-diaglycerides showed that the content of 1,3-diglycerides was rapidly increased to 36.90% with 25 min of ultrasound duration (*P* < 0.05) and later decreased to 19.93% with 35 min of ultrasound duration. The modeled structure of PEXANL1 by comparative modeling showed α/β hydrolase fold. Structural superposition and molecular docking results validated that Ser162, His274, and Asp217 residues of PEXANL1 were involved in the catalysis. Small-angle X-ray scattering analysis indicated the monomer properties of PEXANL1 in solution. The *ab initio* model of PEXANL1 overlapped with its modeling structure. This work presents a reliable structural model of *A. niger* lipase based on homology modeling and small-angle X-ray scattering. Besides, the data from this study will benefit the rational design of suitable crystalline lipase variants in the future.

## Introduction

Lipase (EC 3.1.1.3), an important class of hydrolytic enzymes, is involved in the hydrolysis of medium and long-chain triglycerides. Interfacial activation is a unique feature of lipase. In this process, lipase could rapidly catalyze the hydrolysis of insoluble lipids on the oil-water interface (Ferrato et al., 1997). High regional- and stereo-selectivity involved in transesterification, acidolysis, and alcoholysis reactions are other unique features of lipases (Antonetti et al., 2020; Utama et al., 2020; Varol et al., 2021). Multiple studies on lipase have been conducted, specifically in microbial lipases (Guldhe et al., 2016). *Aspergillus niger* lipase has good biosafety, and it catalyzes the hydrolysis (or synthesis) of triacylglycerols with sn-1, 3 selectivity when optimal substrate fatty acid carbon chain length is in the range of C6–C12. Therefore, *A. niger* lipase has always been an important food additive in traditional food processing (Liu et al., 2015).

1,3-diaglycerides (1,3-DAG) synthesis is an important application of lipase with sn-1,3 selectivity. 1,3-DAG is a structural oil with multiple health benefits, such as lowering blood lipids, blood pressure, and weight loss (Nicholson and Marangoni, 2019). Glycerolysis and esterification are the primary reactions in sn-1,3 selective lipase-catalyzed 1,3-DAG synthesis, and these reactions can be categorized into the solvent and solvent-free systems (Zhao et al., 2019, 2020). In a solvent system, as the substrate is completely dissolved in the solvent, the contact area of solvent with powered or liquid enzyme increases remarkably than that in a solvent-free system. Although it is one of the advantages of the solvent system, catalytic activity, stability, and selectivity of the enzymes are altered in the solvent system. 1,3-DAG synthesis using ultrasound technology has gained a lot of attention recently due to its multiple advantages, such as effective cavitation energy and significant mixing effects (Abedi et al., 2019). Apart from being an environmentally friendly method, the ultrasonic-assisted enzymatic method could substantially improve the reaction efficiency as against the traditional lipase catalytic method (Chatel, 2017). In lipase-catalyzed reaction, the particle size of the substrate is reduced and substrate-lipase interface area is increased, which is highly conducive for mass transfer; besides, it improves the performance of lipase (Deshmane et al., 2008).

X-ray diffraction, cryo-electron microscopy, and nuclear magnetic resonance are commonly used methods for studying the protein structure, of which crystal X-ray diffraction is the most commonly used. Due to the overly complex crystallization process of *A. niger* lipases, its 3D structure could not be inferred (Shu et al., 2009) so far. Thus, homology modeling of *A. niger* lipases is a good alternative to its crystal structure. However, a reliable structural model using homology modeling can be obtained only when the amino acid sequence to be modeled are more than 30% similar to the template sequence (Petrey et al., 2003).

Till now, multiple *A. niger* lipases with distinct enzymatic properties were isolated, purified, and characterized (Fukumoto et al., 1963; Namboodiri and Chattopadhyaya, 2000; Mhetras et al., 2009; Liu et al., 2015; Li et al., 2018; Chen et al., 2019), revealing differences in their enzymatic properties and protein structure. In our previous study, a sn-1, 3 extracellular lipase gene (GenBank ID: MK592411.1) was cloned from *A. niger* GZUF36 (Xing et al., 2020). *A. niger* GZUF36 lipase amino acid sequence was translated by using Translate tool^1^.

A total of four lipase sequences of the same length (297 amino acids) were obtained when *A. niger* GZUF36 amino acid sequence was queried by Basic Local Alignment Search Tool in National Center for Biotechnology Information (NCBI-BLAST); however, these amino acid sequences were not completely identical (Table 1). When properties of these four lipases were compared, we observed that the differences in individual positions of the amino acids resulted in major differences in their properties (Table 1). In our previous study, the expression and partial properties of *A. niger* GZUF36 lipase in *Escherichia coli* were characterized. However, as the soluble protein content of the lysate supernatant obtained from *E. coli* was exceptionally low, it hampered the crystallization of *A. niger* lipase as a high amount of protein was required for the crystallization.

**TABLE 1:** Comparison of the amino acid sequence and enzymatic properties of different *Aspergillus niger* lipases.

Enzyme sources	Access number	Length of amino acid sequence	Difference sites^a^	Optimal pH	pH stability	Optimum temperature	Temperature stability	References
*A. niger* GZUF36 lipase	QEP18538.1	297	13L, 41A, 113V, 121D	6.5	6–7	35°C	50°C	To be published
*A. niger* F044 lipase	ABG37906.1	297	13L, 41A, 113I, 121G	7.0	2.0–9.0	45°C	≤60°C	Shu et al., 2007
*A. niger* CBE 332.1 lipase	AID68651.1	297	13L, 41A, 113I, 121D	8.0	7.0–10.0	40°C	≤60°C	Qiao et al., 2017
*A. niger* NCIM 1207 lipase	XP_001397501.1	297	13L, 41S, 113I, 121D	2.5	8.0–11.0	50°C	40°C	Mhetras et al., 2009
*A. niger* A733 lipase	ABG73613.1	297	13H, 41S, 113I, 121D	–	–	–	–	–

*^a^NCBI-BLAST on amino acid sequences of A. niger GZUF36 lipase showed there were differences in these sites 13, 41,113, and 121.*

*P. pastoris* is widely used in gene expression studies as it secrets a high amount of protein. *P. pastoris* has become an ideal gene expression system for heterologous protein expression, particularly for eukaryotic proteins. Besides, it has been widely used in industrial enzyme production (Cregg et al., 2000). In the present study, the extracellular lipase gene from *A. niger* GZUF36 (GenBank ID: MK592411.1) was expressed in *P. pastoris*. The enzymatic properties of the recombinant enzyme (PEXANL1) were characterized. Furthermore, the 3D structure modeled of PEXANL1 was analyzed using homology modeling and small-angle X-ray scattering.

## Materials and Methods

### Strains, Plasmids, and Chemicals

The yeast expression plasmid, i.e., pPIC9K, *P. pastoris* strain GS115 competent cells, and *E. coli* strain JM109, were purchased from Sangon (Shanghai, China). T4 DNA ligase and polymerase chain reaction (PCR) reagents were obtained from Sangon (Shanghai, China). Yeast nitrogen base without amino acids, ampicillin, protein molecular weight markers, Coomassie blue reagent, and zeocin was procured from Solibao (Beijing, China). Restriction enzymes, bioSpin PCR purification kit, plasmid Mini Kit I (100), and DNA gel extraction kit (100) were procured from Omega (Norcross, United States). Triolein, monoolein, 1,3-diolein, 1,2-diolein, and oleic acid were obtained from Sigma-Aldrich (United States). Acetonitrile, *n*-hexane, and isopropanol were of chromatography grade. All other chemicals used in this study were of analytical grade unless otherwise stated.

### Cloning and Expression of PEXANL1

Based on our previous study, the lipase gene (GenBank ID: MK592411.1) coding for the residues 1–297 was cloned using *A. niger* GZUF36 sequence (Xing et al., 2020). Later the *EXANL1* gene encoding mature peptide (without signal peptide coding sequence) was integrated into *P. pastoris* GS115 genome, and it was used to synthesize the recombinant enzyme, as described previously (Zhang et al., 2019). The gene sequence containing a coding sequence of 6 Histidine residues at the 3′ end was optimized according to the codon preference of *P. pastoris*. This gene sequence was synthesized by Sangon (Shanghai, China) and later visually inspected on 1% agarose gel. The synthesized gene and the plain plasmid pPIC9K were digested with *Eco*RI and *Not*I simultaneously and ligated using T4DNA ligase at 16°C. The ligated construct, named pPIC9K-*EXANL1*, was transformed into *E. coli* JM109 cells by applying heat shock. Positive clones were sequenced by Sangon (Shanghai, China). The correct recombinant plasmids were obtained via plasmid Mini Kit I (100) extraction and later digested with *Sal*I enzyme overnight. Linearized and non-linearized plasmids were detected through agarose gel electrophoresis. After *Sal*I linearization, pPIC9K-*EXANL1* was transformed into competent *P. pastoris* GS115 cells electrooptically using a multicopy *Pichia* expression kit (Multicopy *Pichia* Expression Kit, Invitrogen France), as per manufacturers instruction. The clones were inoculated in selective Minimal Dextrose Medium plates containing 0–250 μg/mL G418 and cultured at 30°C for 2–3 days. Seven of the positive clones were verified via the PCR amplification of genomic DNA with 5′AOX1 (5′-GACTGGTTCCAATTGACAAGC-3′) and 3′AOX1 (5′-GCAAATGGCATTCTGACATCC-3′) primers.

The seven positive clones were named as A, B, C, D, E, F, and G and they were tested for protein expression. Briefly, these clones were cultured on Yeast Extract Peptone Dextrose (YEPD) plates for 2–3 days, and a single colony from the YEPD plate was inoculated into 25 mL of the buffered glycerol-complex medium and incubated overnight in a 250 mL Erlenmeyer flask at 30°C with a constant shaking of 200 rpm. OD_600_ was measured after overnight incubation. The culture with 4 OD was centrifuged (CR3i multifunction centrifuge; Thermo Electron Co., France; same as below) at 3000 × *g* for 5 min at 4°C. The resulting pellets were resuspended into 100 mL buffered methanol-complex medium and incubated at 30°C for 96 h. The recombinant protein was induced every 24 h by adding 1.0% (v/v) methanol. The fermented broth was centrifuged at 10,000 × *g* for 10 min at 4°C to obtain a crude enzyme solution. This crude enzyme solution was electrophoretically resolved using sodium dodecyl sulfate-polyacrylamide gel electrophoresis (SDS-PAGE), and expression of target proteins were probed using western blot (WB), as described in our previous report (Xing et al., 2020). The clones transferred into the empty plasmid was served as controls.

Based on the outcomes of the electrophoresis analysis, the seventh clone G was selected and cultured as described previously, to optimize its expression conditions. The variables were set to induction time (24, 48, 72, 96, 120, and 144 h) and methanol addition (0.5, 1.0, 1.5, 2, and 2.5%, v/v). The supernatant of fermented broth was periodically removed to determine the optimal conditions of the lipase activity.

### PEXANL1 Protein Purification

Nickel nitrilotriacetic acid (Ni^2+^-NTA) sepharose affinity chromatography was used to purify 6×His-tagged recombinant protein, as described previously (Li et al., 2015). The crude enzyme was dialyzed against buffer A (20 mM Tris-HCl, 500 mM NaCl, 10% glycerol, pH 8.0) overnight. The resulting recombinant protein was filtered through a 0.22 μm filter membrane and concentrated using an ultrafiltration tube with a 10 kDa cut-off (Millipore, United States). The resulting concentrate was injected into an AKTA Prime Plus (GE, Germany) protein purification system equipped with a Ni-NTA affinity chromatography column and equilibrated with buffer A at 1 mL/min. In the washing step, unbound proteins were washed with an imidazole-free buffer at 1 mL/min. Different concentrations of imidazole buffer A (10 and 250 mM) were used for elution at an elution rate of 1.0 mL/min. To remove imidazole from the eluted active component, it was dialyzed against 20 mM Tris-HCl buffer (pH 8.0) overnight at 4°C. The concentration of purified protein was determined using the Pierce BCA protein assay kit (Smith et al., 1985), and bovine serum albumin was used as the standard. The purified protein was resolved on SDS-PAGE.

Deglycosylation of lipase was performed by incubating the purified enzyme (5 mg/ml) with 1,000 U/μL of endoglycosidase Hf (Endo Hf, New England Biolabs) at 37°C for 2–24 h, as per the manufacturer’s instruction. The deglycosylated enzyme was visually inspected by resolving it on SDS-PAGE. Endo Hf with maltose-binding protein fusion tag specifically binds to amylose resin. Thus, the solution containing deglycosylated enzyme was applied to amylose resin pre-equilibrated with 20 mM Tris buffer containing 150 mM NaCl, 1 mM EDTA (pH 7.4) to remove Endo Hf. Unabsorbed protein was eluted as the purified deglycosylated enzyme and analyzed using SDS-PAGE.

Finally, the purified deglycosylated enzyme was concentrated to 5 mg/mL by using an ultrafiltration tube with 10 kDa cut-off (Millipore, United States), and concentrated protein was stored at −20°C for further analysis.

### Lipase Activity Assay

Alkali titration method was used to determine the hydrolytic activity of PEXANL1 using olive oil as substrate, as described previously (Chen et al., 2019). Briefly, olive oil was emulsified in 4% (w/v) polyvinyl alcohol solution at a ratio of 1: 3 (v/v). The reaction mixtures contained 4 mL of emulsified olive oil, 5 mL of 50 mM disodium hydrogen phosphate-citrate buffer (pH 4.0), and 1 mL of appropriately diluted enzyme solution and incubated at 40°C. The reaction was terminated by using 15 mL of 95% ethanol. The fatty acids released were neutralized with 0.05 M NaOH. One unit was defined as the amount of enzyme required to release 1 μmol of titratable fatty acid per minute under the assay conditions.

### Biochemical Characterization of PEXANL1

#### Effect of pH and Temperature on the Enzymatic Activity of PEXANL1

The effect of pH on the PEXANL1 activity was examined by emulsifying the substrate (olive oil) at 40°C in two distinct 50 mM buffer, i.e., sodium citrate-sodium phosphate (pH 2.0–8.0) and sodium carbonate (pH 9.0–10.0). To gauge the pH stability, PEXANL1 was pre-incubated in these buffers (1: 2 v/v) at 4°C for 48 h. The lipase activity was determined using olive oil as substrate at 40°C, as per the lipase assay mentioned in Section “Lipase Activity Assay.” The effect of temperature on the lipase activity was investigated by dissolving PEXANL1 in 50 mM of sodium citrate-sodium phosphate buffer (pH 4.0) with olive oil (PEXANL1 substrate) emulsification. This reaction was carried out at a temperature gradient of 20–65°C. To determine the thermal stability, PEXANL1 in 50 mM sodium citrate sodium phosphate buffer (pH 4.0) was incubated at a temperature range of 45–60°C for 60 min. The aliquots were withdrawn at every 15 min intervals, and lipase activity was determined, as per the lipase assay mentioned in Section “Lipase Activity Assay.” The PEXANL1 activity at 40°C and 50 mM of sodium citrate-sodium phosphate (pH 4.0) was considered 100%.

#### Effect of Organic Solvents and Surfactants on Enzyme Activity

20% (v/v) of organic solvents: dichloromethane, *n*-hexane, acetone, acetonitrile, methanol, glacial acetic acid, isopropanol, toluene, tetrahydrofuran, and DMSO, were used to analyze the effect of organic solvents on the PEXANL1 activity. PEXANL1 was pre-incubated with these organic solvents for 2 h at 4°C, and its activity was determined, as per the lipase activity assay mentioned in section “Lipase Activity Assay.” 0.01% (w/v) and 0.1% (w/v) surfactants: Triton X-100, Tween 20, Tween 80, SDS, were employed to determine the effect of surfactants on PEXANL1 activity. PEXANL1 was pre-incubated with these surfactants for 2 h at 4°C, and its activity was determined, as per the lipase activity assay mentioned in Section “Lipase Activity Assay.” The activity of untreated lipase was defined as 100%.

#### Effect of Various Metal Ions on Enzyme Activity

5 mM metal ions (Fe^2+^, Mg^2+^, Zn^2+^, Mn^2+^, Cu^2+^, Ni^2+^, Ca^2+^, K^+^, Na^+^, and Fe^3+^) were added to the PEXANL1 enzyme solution made using 50 mM disodium hydrogen phosphate–citrate buffer (pH 4.0) and incubated for 2 h at 4°C. PEXANL1 activity was determined, as per the lipase activity assay mentioned in Section “Lipase Activity Assay.” The activity of untreated lipase was defined as 100%.

### Ultrasound-Assisted Esterification for 1,3-Diaglyceride

Solvent-free lipase-catalyzed ultrasound-assisted esterification for 1,3-diaglyceride (1,3-DAG) was performed using a reaction mixture containing 0.28 g glycerol and 0.17 g oleic acid in 5: 1 molar ratio, and PEXANL1 to substrate (oleic acid) in 10: 100 mass ratio, in a 5 mL centrifuge tube. This reaction mixture was vortexed and esterification reaction temperature was regulated using a 40°C water bath. The ultrasonic power (200 W) and frequencies (40 kHz) were maintained, and distinct ultrasound duration (5, 10, 15, 20, 25, and 30 min) were studied. For comparative study, the esterification reaction was also used in the traditional stirring method at 180 r/min for 12 h.

### High-Performance Liquid Chromatography Analysis of Glyceride Composition

The composition of glycerides in the esterification reaction mixture of glycerol and oleic acid was analyzed as described previously by Zhong et al. (2012) with slight modification in high-performance liquid chromatography (HPLC) analysis. The sample was prepared by mixing 100 μL of the melted reaction mixture with 900 μL of *n*-hexane. The resulting mixture was filtered through a 0.22 μm nylon membrane to remove impurities. 10 μL of the filtered sample was injected into a Nova-Pak chromatographic column (length 150 mm, inner diameter 3.9 mm, Waters) using an HPLC pump equipped with a diode array detector (Agilent 1260) at 1 mL/min, and the column temperature was maintained at 35°C. The gradient elution conditions are shown in the Supplementary Table 1. The retention time of the peaks in HPLC analysis was compared with commercial standards. DAG content and the conversion rate of oleic acid were calculated using an external standard method. The reaction involved in enzymatic esterification is reversible and involves acyl migration resulting in the formation of products, such as monoglyceride (MAG), 1,3-diglyceride (1,3-DAG), 1,2-diglyceride (1,2-DAG), triglyceride (TAG), and possibly oleic acid (OA). The conversion rate (%) of OA in the sample was calculated as follows: concentration of reduced OA/concentration of starting OA × 100. The 1,3-DAG content in the sample (%) was calculated as follows: 1,3-DAG content/total content of each product × 100.

### Homology Modeling, Refinement, and Quality Assessment of PEXANL1 Modeling Structure

The 3D structure of PEXANL1 was constructed using Modeller version 9.18 (Webb and Sali, 2014) based on comparative modeling with multiple templates. The homologous lipase sequences of *Rasamsonia emersonii* (PDB ID: 6UNV), *Thermomyces lanuginosus* (PDB ID: 1DT3), *Penicillium camemberti* (PDB ID: 1TIA), *Penicillium cyclopium* (PDB ID: 5CH8), *Aspergillus oryzae* (PDB ID: 5XK2), and *Fusarium graminearum* (PDB ID: 3NGM) were used as templates. Furthermore, these sequences were aligned (multiple sequence alignments) using the “salign()” command. Ten independent PEXANL1 models were established using automodel commands, and Discrete Optimized Protein Energy (DOPE) score was used to evaluate these models.

The secondary structure of PEXANL1 based on sequences was predicted via the PsiPred server^2^. 2Strucserver^3^ was also used to analyze the secondary structure of the best PEXANL1 model.

Swiss-PdbViewer^4^ was used to minimize the structural energy of the PEXANL1 model. PEXANL1 modeling structure with the lowest DOPE score was generated using the GROMOS 43B1 force field (Dittrich et al., 2015). The energy-minimized structure of PEXANL1 was validated using the Lagrange diagram (Laskowski et al., 1993), Verify-3D (Colovos and Yeates, 1993), and ERRAT (Lüthy et al., 1992) in the Structural Analysis and Verification Server (SAVES)^5^ on the MBI-UCLA (United States). Statistical Z-score (Wiederstein and Sippl, 2007) of the predicted model, high-resolution crystal structure stored in the PDB database, and protein structure acquired through NMR were calculated through ProSA-web server^6^. The energy minimized PEXANL1 structure, and its homologous lipase structure was superimposed, and amino acid residues in the active site and the lid domain were represented using PyMoL software^7^.

### Molecular Docking Analysis of PEXANL1

AutoDock version 4.2.6 (Morris et al., 2009) was used to access the MGL tool (version 1.56) for molecular docking of PEXANL1. The energy minimized PEXANL1 open-lid modeling structure was used in the above-mentioned docking studies. Diethyl Hydroxymethyl Phosphate (DEP) and tricaprylin were used as ligands for molecular docking study. Ligand files were fetched from the PubChem database^8^ and converted into PDB files using OpenBabel software version 2.3.2a (O’Boyle et al., 2011). Non-polar hydrogen was merged, Gasteiger charge was distributed to the PEXANL1 and ligand PDB files and later saved as PDBQT files. The *x*-, *y*-, and *z*- coordinates of the grid box center (within the active center) was set to 14.38, 45.24, and 5.75, respectively, the size of the grid box was set to 40, 40, and 40 with the default grid point spacing of 0.375 Å. Docking simulation and conformation search were performed using the Lamarckian Genetic Algorithm (LGA). 100 cycles of GA were used for generating 100 distinct ligand confirmations, which were subsequently sorted using binding energy. The best confirmation with the lowest binding energy was used to generate protein-ligand complexes. These protein-ligand complexes are present in PyMOL^9^. 2D interaction of protein and ligand was obtained using Ligplot^+^ software (Laskowski and Swindells, 2011).

### Small-Angle X-Ray Scattering Analysis (SAXS) of PEXANL1

To explore the global shape of PEXANL1, the PEXANL1 SAXS data set was fetched using the SAXS system of the BL19U2 line station (National Facility for Protein Science in Shanghai, NFPS, Shanghai, China) (Liu et al., 2018). The PEXANL1 SAXS intensity was recorded using a 1D CMOS hybrid pixel detector (Dectris, Baden, Switzerland). The distance between the sample and the detector was 26.86 cm. The PEXANL1 concentration of 1, 3.5, and 7 mg/mL was stored in 50 mM Tris-HCl buffer (pH 8.0). The momentum transfer (q) in the range of 0.16–4.2 nm^–1^ was detected (*q* = 4πsinθ/λ, where 2θ is the scattering angle). Before collecting SAXS data, PEXANL1 samples and buffers were centrifuged at 25,000 × *g* for 45 min at 4°C. The wavelength of the incident radiation on the line collimation system was set to 0.9184 A°. PEXANL1 sample and the matched buffer (50 mM Tris-HCl buffer, pH 8.0) filled in a quartz capillary with a diameter of 1 mm was exposed to 0.9184 A° wavelength for 1 s. The resulting scattering pattern (1D image) was integrated using Fit2d software (Hammersley, 2016) to obtain the I(q)-q scattering intensity curve (commonly displayed in double logarithmic coordinates). Twenty scattering images were collected per sample due to radiation damage. To obtain the absolute intensity, an average of the scattering data of the samples and the corresponding buffers was calculated, and background noise was subtracted using the PRIMUS program (Konarev et al., 2003) in ATASA (version 3.0). It validated the dispersion and aggregation behavior of the particles. The Guinier equation and inverse Fourier transform methods in the Primus package estimated the radius of gyration (Rg) of different samples in the Primus package. GNOM package (Semenyuk and Svergun, 1991) was used to calculate the distance distribution function *P*(*r*) and the maximum particle size Dmax of PEXANL1. The PEXANL1 molecular weight was determined using the SAXSMoW program (Fischer et al., 2009). The *ab initio* algorithm in the DAMMIF program was used to reconstruct 20 low-resolution envelopes with a momentum transfer in the range of 0.16–3.6 nm^–1^. These envelopes were aligned and averaged using DAMAVER (Volkov and Svergun, 2003). The SUPCOMB program was used to superimpose and compare the envelopes as well as the models obtained from homology modeling (Kozin and Svergun, 2001).

### Statistical Analysis

All experiments were repeated at least three times. Data are shown as the mean ± standard deviation. ANOVA was performed using SPSS version 20.0 (SPSS, Chicago, IL, United States), and the differences were determined via Tukey’s HSD test and considered statistically significant at *P* < 0.05.

## Results and Discussion

### Cloning and Expression of an sn-1,3 Lipase Gene From *A. niger* GZUF36

An *A. niger* GZUF36 sn-1,3 extracellular lipase gene, *EXANL1* (GenBank ID: MK592411.1), was successfully cloned and transformed into *P. pastoris* GS115 competent for recombinant EXANL1 (named as PEXANL1). The gene has an open reading frame (ORF) of 894 bp, encoding 297 amino acids long protein (mature peptide as 278) with a theoretical molecular mass of 31.7 kDa and a theoretical pI of 4.47, as per the Protparam server^10^ analysis. The amino acid sequence alignment of *EXANL1* encoding a deduced protein with several known structures for lipases indicated that the encoded enzyme belonged to the alpha-beta hydrolase superfamily 23 (abH23)-filamentous fungi lipases. PEXANL1 exhibited the highest sequence identity of 54.04% with the characterized lipase from *R. emersonii* [PDB ID: 6UVN (Rade et al., 2020)], followed by 50.19% sequence identity with lipases from *T. lanuginosus* (PDB ID: 1DT3) (Brzozowski et al., 2000), 43.33% with *P. camemberti* (PDB ID: 1TIA) (Derewenda et al., 1994), 43.33% with *P. cyclopium* (PDB ID: 5CH8) (Tang et al., 2016), 40.57% for *A. oryzae* (PDB ID: 5XK2), 39.63% with *F. graminearum* (PDB ID: 3NGM) (Lou et al., 2010), 34.93% with *Rhizomucor miehei* (PDB ID: 5TGL, 34.93%, (Brzozowski et al., 1991) and 38.18% with ferulic acid esterase from *A. niger* (PDB ID: 1USW) (Hermoso et al., 2004). The multiple sequence alignment (MSA) was performed using CLUSTALW (Larkin et al., 2007) and ESPript 3 software (Robert and Gouet, 2014). The results showed that PEXANL1 contained conserved, semi-conserved, and dispersive amino acid residues. The conserved residues were highlighted with red color background, and semi-conserved residues were highlighted with red color (Supplementary Figure 1).

MSA of PEXANL1 showed 11 α-helix (7α + 4η), 10 β-sheets, and 5 turns (TT) in PEXANL1. Y37, F96, R97, G98, N105, G128, G160, H161, S162, L163, G164, L165, P190, R191, A198, R211, D217, P220, L223, P224, G228, E235, Y236, H274, Y277, and F278 were some of the conserved amino acids in PEXANL1 as per MSA. S162-D217-H274 conserved and catalytic residues are represented by a triangle. The amino acid sequence corresponding to the lid domain is represented by a square box located behind the FRG conserved motif, and green number pairs represent disulfide bonds (Supplementary Figure 1).

**FIGURE 1:**
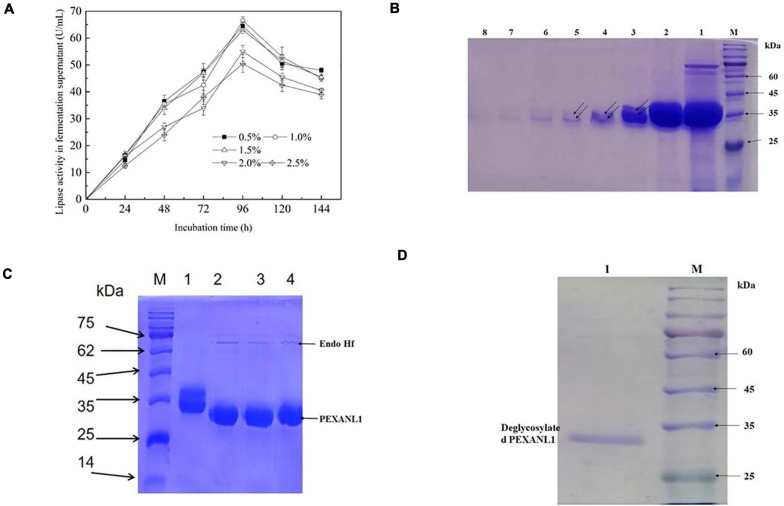
Optimal expression conditions and gel electrophoresis of purification process of PEXANL1. **(A)** The effect of methanol addition and induction time on PEXANL1 produced by *Pichia pastoris* GS115. The enzyme activity of the yeast-induced fermentation supernatant was measured periodically. **(B)** SDS-PAGE analysis of PEXANL1 purified by a Ni-NTA column. Lane M: The molecular weight marker. Lane 1: Crude enzyme ultrafiltration concentrate; lanes 2–8: 3 mL of buffer A elution collection solution containing 250 mM imidazole, corresponding to 1–7 tubes, the double arrow pointed to the two molecular weights of PEXANL1 due to the difference in sugar content. **(C)** SDS-PAGE analysis of deglycosylation of purified PEXANL1. Lane M: The molecular weight marker. Lane 1: Purified PEXANL1; lanes 2–4: purified PEXANL1 treated with Endo Hf. **(D)** SDS-PAGE analysis of removal of Endo Hf by a amylose resin column. Lane M: the molecular weight marker. Lane 1: deglycosylated purified PEXANL1 showing a single band around 35 kDa.

The gene sequence encoding 6 Histidine residues at 3′ end was inserted into the multiple cloning sites of pPIC9K and named as pPIC9K-*EXANL1.* Electropherogram showed linearization of pPIC9K-*EXANL1* plasmid (Supplementary Figure 2A). This linear plasmid was later electrotransformed into *P. pastoris* GS115 competent cells. The selected seven positive strains A, B, C, D, E, F, and G were validated using PCR, and results showed that PEXANL1 was successfully integrated into the *P. pastoris* genome (Supplementary Figure 2B). 1.0% (v/v) methanol was used to induce these positive strains, and later PEXANL1 expression was analyzed. As per the electrophoresis results, the fermentation supernatant of the positive strains A, B, C, D, E, F, and G all showed a clear and deep band but diffused near the theoretical molecular weight (31.7 kDa) as compared to the fermentation supernatant of the negative strain (Supplementary Figure 2C). WB based validation analysis of the fermentation supernatant of the positive strains A, B, C, D, E, F, and G all showed a dispersion band near the corresponding molecular weight (Supplementary Figure 2D). It indicated successful expression of PEXANL1. Among the positive strains, 100 mL BMMY supernatant of the strain G represented the highest activity of 17 ± 2.3 U/mL was observed (data not shown). Furthermore, the culture conditions of the strain G were also optimized. The addition amount of methanol and the induction time showed a significant effect on PEXANL1 lipase activity (Figure 1A). Addition of 0.5–1.5% (v/v) of methanol induced a higher lipase activity in fermentation supernatant as compared to 0.5–1.5% (v/v) of methanol. However, the highest lipase activity was induced by 0.1% (v/v) of methanol. Lipase in fermentation supernatant started accumulating as the induction time increased, with an apparent increase in lipase activity. However, with prolonged cultivation, lipase activity began to decline, which might have led to degradation or partial inactivation of the accumulated lipase.

Methanol amount and induction time were optimized, and for the highest PEXANL1 activity of 66.5 ± 1.4 U/mL, 96 h was found to be the optimum induction time and 1.0% (v/v) methanol as optimum methanol concentration. In our previous study, this lipase was expressed in *E. coli* and the maximum lipase activity from the cell lysate without concentration was only 1.2 ± 0.13 U/mL under optimal conditions at 16°C and for 16 h induction (data are not shown). In this study, 55 times higher activity of *A. niger* GZUF36 lipase expressed in *P. pastoris* was observed as compared to the previous study in *E. coli*. We speculate that since the rate of correct folding is lower than the rate of synthetic peptides, most of the *A. niger* GZUF36 lipase expressed in *E. coli* forms misfolded aggregates, namely inclusion bodies (Villaverde and Carrió, 2003). Thus, *P. pastoris* is a more appropriate expression host for eukaryotic proteins, especially for *A. niger* lipase.

### Purification of PEXANL1

PEXANL1 protein was purified using nickel affinity chromatography. PEXANL1 was 12.8-fold purified with a 64.1% increase in yield, resulting in 206.8 U/mg specific activity (Supplementary Table 2). Two bands were observed in the SDS-PAGE analysis of purified PEXANL1 (Figure 1B). A higher concentration of PEXANL1 resulted in slightly diffuse bands and a lower PEXANL1 concentration in sharp bands. The molecular weight for one of the bands was ∼35 kDa, and the other presented a slightly higher molecular weight of 35–40 kDa. Molecular weights of these two bands containing 6×His tag were higher than the estimated value of 31.5 kDa. This discrepancy might be due to the potential glycosylation of PEXANL1. It is also well known that *P. pastoris* can add O-linked and N-linked carbohydrates moieties to secreted proteins (Macauley-Patrick et al., 2010). As per the analysis of online tool Prediction Servers NetNGlyc version 1.0^11^, three putative N-linked glycosylation sites (^48^NVTC, ^139^NLTS, and ^258^NSTA) were predicted in the PEXANL1. The amino acid sequence of PEXANL1 was different from the previously reported amino acid sequence of *A. niger* lipase due to the different positions of amino acids (Table 1). These lipases expressed in *P. pastoris* showed two lipase bands due to glycosylation. Therefore, we believed that the two bands of purified PEXANL1 were lipase bands, and the difference in molecular weight of these bands was due to the difference in sugar content.

To remove sugar from purified PEXANL1, it was treated with Endo Hf. As per the outcomes, Endo Hf treatment led to a downward shift of the PEXANL1 bands on SDS-PAGE as compared to untreated protein (Figure 1C). The results validated glycosylation modification in PEXANL1. As Endo Hf was a glycosidase fused with maltose-binding protein, deglycosylated samples were passed through amylose resin to remove Endo Hf. As shown in Figure 1D, the homogeneous protein was obtained after the removal of Endo Hf.

### Biochemical Characterization of PEXANL1

#### Optimum pH and pH Stability of PEXANL1

**FIGURE 2:**
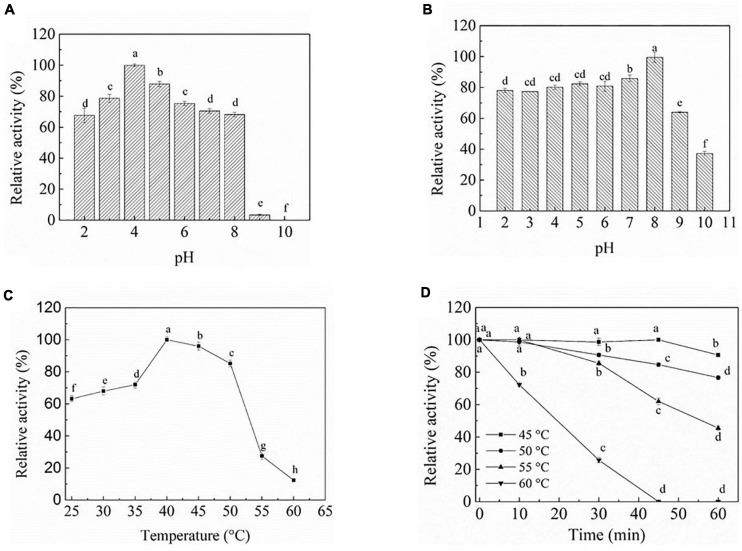
Biochemical characterization of PEXANL1. **(A)** The pH optimum. The activity was determined at pH 2.0–10.0 and 40°C using olive oil as the substrate. 100% of activity corresponds to 206.8 ± 3.47 U/mg, the same behind. Means with dissimilar lower case letters indicate significant differences between groups (*P* < 0.05), the same behind. **(B)** pH stability of purified PEXANL1. The remaining activity was determined at 40°C and pH 4.0 after incubated in pH 2.0–10.0 50 mM buffer for 48 h. **(C)** The temperature optimum. The activity was determined at 25–60°C and pH 4 using olive oil as the substrate. **(D)** Thermal stability of purified PEXANL1. The remaining activity was determined at 40°C and pH 4.0 after incubated for 60 min at 45–60°C.

To evaluate the effect of pH on PEXANL1 activity, two distinct buffers with a wide pH range of 2–10 (50 mM disodium hydrogen phosphate-citrate buffer, pH 2–8; 50 mM carbonate buffer, pH 9–10) were used. The outcomes of this analysis are shown in Figure 2A. PEXANL1 showed >70% activity in the pH range of 3–8, and the highest activity of 100% (206.8 ± 3.47 U/mg) was observed at pH 4 (*P* < 0.05). However, the PEXANL1 activity decreased significantly at pH > 8 (*P* < 0.05). Besides, the structure and activity of PEXANL1 were destroyed in a high pH range (pH > 8). It indicated that PEXANL1 needs to attain an appropriate ionic form to maintain its active site conformation to bind to its substrate properly.

The PEXANL1 stability was evaluated at a wide pH range of 2–10 (50 mM disodium hydrogen phosphate-citrate buffer, pH 2–8; 50 mM carbonate buffer, pH 9–10). The results of this analysis are depicted in Figure 2B. PEXANL1 retained about 80% of its enzymatic activity when incubated at pH 2.0–8.0 for 48 h. When stored at pH 8.0 for 48 h, 100% of the PEXANL1′ activity was retained (*P* < 0.05). However, the residual rate of PEXANL1’ activity reduced significantly at pH > 8.0 (*P* < 0.05; Figure 2B). Interestingly, PEXANL1 retained around 100% of its activity when stored for 1 week at pH 8.0; conversely, PEXANL1 activity dropped down to 10% when stored at pH 4.0 for 1 week (data not shown). A significant difference was observed between the optimum pH and pH stability of PEXANL1. As per the previous reports (Esakkiraj et al., 2017; Eddehech et al., 2019), acidic amino acids (negatively charged) on the enzyme surface imparts stability to the enzymes, and these amino acids are stable at alkaline or basophilic conditions. This explains the high stability of PEXANL1 at pH 8.0. As stated previously, *A. niger* NCIM 1207 lipase, which has an optimal pH of 2.5, was stable at pH 8–11 (Mhetras et al., 2009).

#### Optimum Temperature and Temperature Stability of PEXANL1

The effect of temperature (25–60°C) on PEXANL1 activity was evaluated, as shown in Figure 2C. More than 70% increase in PEXANL1 activity was observed at a temperature range of 35–50°C, and the highest PEXANL1 activity was observed at a temperature of 40°C (*P* < 0.05). However, a temperature higher than 55°C significantly reduced the PEXANL1 activity. PEXANL1 exhibited maximal activity at a temperature range of 35–50°C. It indicated that PEXANL1 belonged to the medium-temperature lipase group.

PEXANL1 was exposed to a wide temperature range of 45–60°C to investigate the activity and thermostability of PEXANL1 (Figure 2D). 90% of the PEXANL1 enzyme activity was retained when PEXANL1 was incubated at 45°C for 60 min. It showed that the PEXANL1 was stable at 45°C as well as 50°C. When incubated at 55°C for 60 min, around 55% of PEXANL1 activity was retained (*P* < 0.05). Conversely, when incubated at 60°C for 30 min, the residual activity of PEXANL1 was only 20% (*P* < 0.05), which indicated that the PEXANL1 was not tolerant to temperature >55°C. Most *Aspergillus* lipases are not resistant to high temperature, and only few lipases, such as *Aspergillus terreus* lipase, were tolerant to high temperature (70°C) (Yadav et al., 2011).

#### Comparative Analysis of the Basic Enzymatic Properties of PEXANL1, Lipase Expressed in *E. coli* and Native Lipase From *A. niger* GZUF36

We summarized the enzymatic properties of *A. niger* GZUF36 extracellular lipase and its heterologously expressed recombinant enzymes in terms of the optimum pH, pH stability, optimum temperature, and temperature stability. According to Supplementary Table 3, we found that the basic properties of the lipase from the heterologous expression of the *A. niger* extracellular lipase (*E. coli* and *P. pastoris*) were almost the same, except for the optimum temperature. We speculated recombinant enzymes expressed in *P. pastoris* were more easily *N*-glycosylated by post-translational modification than that expressed in *E. coli*. PEXANL1 has been deglycosylated and its protein core structure should be exactly the same as the recombinant enzyme expressed in *E. coli* (without glycosylation modification). Therefore, the difference in the optimal temperature between the PEXANL1 and the recombinant enzyme expressed in *E. coli* may be due to the difference in the N-terminal structure produced by the expression strategy. The N-terminal structure may affect the catalytic performance of the enzyme (Huang et al., 2019).

However, It was obvious that the basic properties of the PEXANL1 and the recombinant enzyme expressed in *E. coli* both were very different from the native lipase. The reason may be that the native lipase has glycosylation modification (Chen et al., 2019). Thermal stabilities of endo-1, 4-b-D-glucanases can be decreased or increased after glycosylation (Zhao et al., 2012). The activity and enzymatic properties of PEXANL1, lipase expressed in *E. coli* and native lipase was possibly affected due to different sources of expression and differences in the degree of glycosylation modification, sugar content and sugar types.

#### Effect of Organic Solvents and Surfactants on PEXANL1 Activity

The tolerance of enzymes to organic solvents is an important issue in the chemical industry. Hence, the effect of multiple polar and non-polar organic solvents on the PEXANL1 activity were investigated. As shown in Table 2, a higher Log*P* value of solvents indicated higher PEXANL1 residual activity. In other words, the maintained activity of PEXANL1 was higher in non-polar solvents, specifically in *n*-hexane (high Log*P* value), the maintained activity of PEXANL1 was 106.02 ± 1.05%, but there were certain exceptions. For instance, the residual activity of the PEXANL1 enzyme in dichloromethane (non-polar solvent) was 0, whereas in dimethyl sulfoxide (polar solvent) it was 88.86 ± 1.10%. The PEXANL1 activity increased by 6% when incubated with 20% (v/v) *n*-hexane at 4°C for 2 h. It was an important finding since *n*-hexane is commonly used in multiple lipase-catalyzed reactions, such as the production of biodiesel, flavored products, and pharmaceutical products. However, dichloromethane inactivated PEXANL1. It suggested that dichloromethane might have modified the N-terminus amino-acid residues of PEXANL1, damaging the spatial structure of PEXANL1, culminating in its inactivation. Glacial acetic acid led PEXANL1 inactivation might be due to extremely low pH and enzyme surface polarization, which might have made the active site of the enzyme inaccessible to the substrates.

**TABLE 2:** Effect of various organic solvents on the activity of PEXANL1.

Organic solvents (20%, v/v)	Log*P*^a^	Relative activity (%)^b,c^
Control		100.00 ± 1.15^b^
Ice acetic acid	–0.23	0^i^
Methylene chloride	1.4	0^i^
*n*-hexanol	1.4	76.70 ± 1.88^f^
Dimethyl sulfoxide	–1.3	88.86 ± 1.10^d^
Tetrahydrofuran	0.49	84.58 ± 1.78^e^
*n*-hexane	3.5	106.02 ± 1.05^a^
Acetonitrile	–0.33	89.65 ± 0.29^d^
Toluene	2.5	95.95 ± 0.26^c^
Isopropanol	0.16	50.65 ± 3.38^h^
Methanol	–0.76	84.41 ± 3.38^e^
Acetone	–0.23	61.73 ± 0.26^g^

*^a^Log Pow (LogP) is the logarithm of the partition coefficient of a solvent between n-octanol and water, used as a quantitative measure of solvent polarity. ^b^The enzyme was incubated 2 h at 4°C in the presence of solvent (20%, v/v), and then the residual activity was determined at pH 4 and 40°C using olive oil as substrate. The control was measured after incubation of the enzyme in absence of organic solvent. ^c^Values (mean ± standard deviation) with different lower case letters in a column indicate significant differences among tested organic solvents (P < 0.05).*

Surfactants play a crucial role in the emulsions, which are widely used in lipase assays and characterization. In this study, we investigated the effect of surfactants (Triton X-100, Tween 20, Tween 80, and SDS) on the PEXANL1 activity. Triton X-100, Tween 20, Tween 80, and SDS (0.01% w/v) enhanced the PEXANL1 activity 2 h post-incubation at 4°C (Table 3). In particular, PEXANL1 activity was increased to 118% when it was incubated with Tween 20 (0.01% w/v) for 2 h at 4°C. However, the PEXANL1 activity was significantly inhibited by 0.1% w/v of Triton X-100, Tween 80, and SDS, of which 0.1% Triton X-100 showed the most inhibitory effects on PEXANL1 activity. Interestingly, Tween 20 (0.1% w/v) increased the PEXANL1 activity by 38%.

**TABLE 3:** Effect of surfactants on the activity of PEXANL1.

Surfactants	Residual activity (%) (0.01%, w/v)^a,b^	Residual activity (%) (0.1%, w/v)
Control	100.00 ± 2.54^c^	100.00 ± 2.54^b^
Triton X-100	106.22 ± 1.72^b^	0^e^
SDS	106.82 ± 0.77^b^	9.85 ± 0.83^*d*^
Tween 20	118.51 ± 1.18^a^	137.76 ± 0.91^a^
Tween 80	109.40 ± 0.63^b^	94.28 ± 0.23^c^

*^a^The enzyme was incubated 2 h at 4°C in the presence of surfactants (0.01%, v/v) and (0.1%, v/v), and then the residual activity was determined at pH 4 and 40°C using olive oil as substrate. The control was measured after incubation of the enzyme in absence of surfactants. ^b^Values (mean ± standard deviation) with different lower case letters in a column indicate significant differences among tested surfactants for the same concentration (P < 0.05).*

#### Effect of Metal Ions on the Activity of PEXANL1

Metal ions react with disulfuric acid in the solution and significantly affect enzymatic activity by changing enzyme structure or enzyme-substrate reaction rate. In this study, all-metal ions tested at a final concentration of 5 mM did not activate PEXANL1; however, Fe^3+^ metal significantly inhibited PEXANL1 (Table 4). Notably, the results also showed that PEXANL1 did not depend on Ca^2+^ activation; besides, Ca^2+^ inhibited the lipase activity (75.00% ± 0.00%). However, Ca^2+^ promotes the activity of most lipases, especially Ca^2+^-dependent lipases, which require Ca^2+^ for activation (Yang et al., 2010).

**TABLE 4:** Effect of metal ions on the activity of PEXANL1.

Metal ions (5 mM)	Relative activity (%)^a,b^
Control	100.83 ± 1.44^a^
Zn^2+^	76.83 ± 2.10^f^
Cu^2+^	84.75 ± 2.38^e^
Na^+^	87.96 ± 0.79^c^
Mn^2+^	76.83 ± 2.10^d^
Ca^2+^	75.00 ± 0.24^d^
K^+^	88.88 ± 2.38^c^
Ni^2+^	90.92 ± 1.23^b^
Fe^3+^	65.71 ± 1.59^g^
Fe^2+^	75.00 ± 0.12^d^
Mg^2+^	87.96 ± 0.79^c^

*^a^The enzyme was incubated 2 h at 4°C in the presence of metal ions (5 mM), and then the residual activity was determined at pH 4 and 40°C using olive oil as substrate. The control was measured after incubation of the enzyme in absence of metal ions. ^b^Values (mean ± standard deviation) with different lower case letters in a column indicate significant differences among tested metal ions (P < 0.05).*

### Ultrasound-Assisted PEXANL1 Esterification for 1,3-Diaglyceride

**FIGURE 3:**
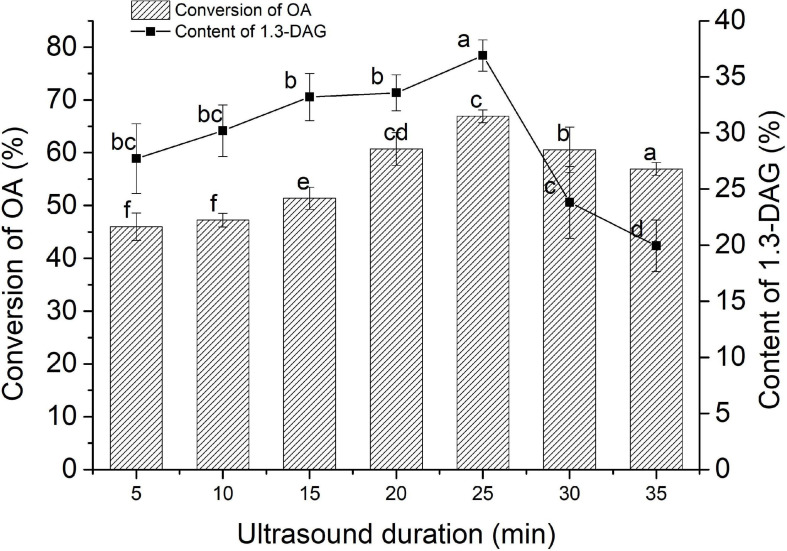
The effect of ultrasound duration on the synthesis of 1,3-DAG by PEXANL1. Lipase-catalyzed esterification reaction conditions: 5:1 molar ratio of glycerol to oleic acid, 40°C temperature, the ultrasonic power (200 w) and frequencies (40 kHz), and ultrasound duration (5, 10, 15, 20, 25, 30, and 35 min). Means with dissimilar lower case letters on the curve or bar indicate significant differences among ultrasound duration (*P* < 0.05).

Ultrasound-assisted esterification of oleic acid (OA) with glycerol was studied for 1,3-diaglyceride (1, 3-DAG) synthesis using PEXANL1. The effect of different ultrasound durations (5, 10, 15, 20, 25, 30, and 35 min) on 1, 3-DAG production was investigated (Figure 3). The 1, 3-DAG content rapidly increased to 36.90% with 25 min of ultrasound duration (*P* < 0.05) and later decreased to 19.93% with 35 min of ultrasound duration. OA conversion was rapidly increased to 66.90% with 25 min of ultrasound duration (*P* < 0.05) and later decreased to 56.93% with 35 min of ultrasound duration.

The traditional stirring method on 1, 3-DAG production at 180 r/min for 12 h was also studied. 1, 3-DAG content and OA conversion with the traditional stirring method was found to be 33.68 and 69.07%, respectively. The results indicated that the time for the 1,3-DAG production using ultrasound-assisted lipase-catalyzed esterification reaction was substantially reduced. These findings were in line with the study by Gupta et al. (2020), in which ultrasound-assisted reactants were completely miscible with good mass transfer and fast reaction rate. It suggested that appropriate ultrasonic treatment can increase the substrate and the enzyme dispersibility and structure of the enzyme (Huang et al., 2016). The high interface area between the microphases of oleic acid and glycerol could increase the reaction rate and achieve the best conversion at a lower enzyme level (Deshmane et al., 2008; Sivakumar et al., 2014). Thus, ultrasound is a more efficient method for 1,3-DAG synthesis.

### Secondary Structure Analysis of PEXANL1

PsiPred server was used to predict the secondary structure of PEXANL1. As per the outcomes, PEXANL1 secondary structure entailed 27.7% α-helices, 17.8% β-strands, and 54.5% random coils. Besides, 2Struc server, which is used for 3D structure prediction, showed that PEXANL1 contained 32.2% α-helix, 19.5% β-sheet, and 48.3% random coil. Thus, it can be concluded that lipase contained a more significant proportion of alpha-helix secondary structure. The alpha helix of lipases stabilizes protein structure, and the antiparallel beta-sheet of lipases forms the core domain. The catalytic triad in PEXANL1 is located at the junction of the alpha-helix and beta-sheet and embedded inside the alpha helix. The PEXANL1 secondary structure prediction performed using different servers were relatively consistent.

### Homology Modeling and Structure Validation of PEXANL1

**FIGURE 4:**
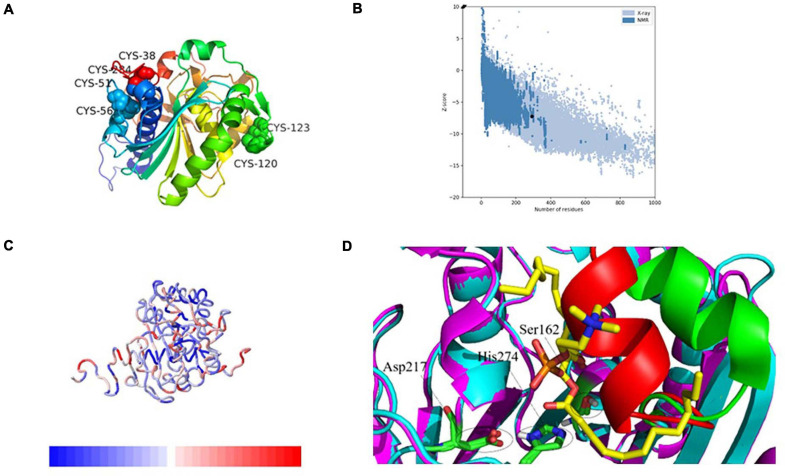
3-Dimensional structure analysis of PEXANL1 by homology modeling and its validation. **(A)** 3D structure (rainbow colors) showing α/β hydrolase fold with three disulfide bonds. **(B)** ProSA plot showed *Z*-score, –6.76 for overall model quality. **(C)** ProSA local energy 3D structure. **(D)** Structure superposition of modeled PEXANL1 (cyan) with TLL in complex with diundecyl phosphatidyl choline (PDB ID: 1EIN) (magenta) showing the binding cleft and the substrate could not be accommodated in the active-site cleft of modeled PEXANL1. Red and green represent the lid of modeled PEXANL1 and the lid of TLL, respectively.

The 3D modeled structure of PEXANL1 displayed classical α/β hydrolase fold. Besides, it consisted of a major eight-stranded mixed β-sheet, two minor two-stranded β-sheet arrangements, and five α-helices (Figure 4A). Based on homologous lipase structures, the modeled PEXANL1 displayed three disulfide bonds (C51–C56, C38–C284, and C120–C123) (Figure 4A). Out of these three disulfide bonds, one disulfide bond (C120–C123, represented as a green ball) was located near the lid subdomain, as was the case with other lipases and esterases. C38–C284 formed the disulfide bridge, which connected the N-terminus and C-terminus. Moreover, this disulfide bond was adjacent to the disulfide bond formed between C51–C56, making the protein structure more compact and stable.

The quality of the energy minimization model structure was also assessed. The Lagrange diagram mainly evaluates the rationality of each amino acid of the model. The model with more than 90% of the amino acid ratio in the favorable region is considered of high quality. As per the Lagrange diagram analysis of the PEXANL1 modeled structure, 90.0% (233 amino acids) of the amino acid residues were present in the most favorable region. Besides, 8.5% (22 amino acid) of the amino acid residues were in the additional allowed region, 0.8% (2 amino acids: Ser 162 and Phe 278) of the amino acid residues in the generously allowed region, and only 0.8% (2 amino acids: Asn 74 and Leu 215) of the amino acid residues were in the disallowed region (Supplementary Figure 3A). It indicated the high quality of the PEXANL1 model.

As per the VERIFY 3D structure, 87.41% of amino acids in the PEXANL1 model showed an overall score of more than 0.2 (Supplementary Figure 3B). ProSA results validated the X-ray zone *Z*-score of −7.24 for the PEXANL1 model, which was close to *Z*-scores of the two templates used in the comparative modeling method (PDB ID: 6UNV, *Z*-score: −5.77; PDB ID: 1DT3, *Z*-score: −8.51) (Figure 4B). The outcomes of the quality assessment were encouraging and prompted further research on PEXANL1. ProSA demonstrated the local energy of each amino acid (blue represents lower energy, red represents higher energy) (Figure 4C). ERRATA plot quality factor of PEXANL1 model structure was 95.41%, which validated the high quality of the PEXANL1 model (Supplementary Figure 3C).

Furthermore, PEXANL1 modeled structure and *T. lanuginosus* lipase structure (PDB ID: 1EIN) were superimposed to obtain the catalytically active ternary structure of PEXANL1. The PEXANL1 catalytic ternary structure consisted of Ser162, His274, and Asp217 residues (Figure 4D). This superposition study suggested that the catalytic ternary structure was conserved and aligned in space.

High lipase activity is realized, by and large, when substrate concentration is identical to that of micellar concentration; however, this catalysis reaction does not follow the Michaelis equation. This interface activation phenomenon is related to the large hydrophobic block formed by the closed conformation lid subdomain of lipase (Skjold-Jørgensen et al., 2014; Khan et al., 2017). When the enzyme identifies the substrate, the lid undergoes a conformational change, and the hydrophobic block of the enzyme is exposed, which interacts with the substrate. Sequence homology revealed that the PEXANL1 model structure (Figure 4D) contained an α-helix lid that was involved in interface activation and substrate recognition similar to *T. lanuginosus’* lipases. Herein, interfacial activation of PEXANL1 could be explained through the superimposed model of PEXANL1 and the available lid-opening structure of *T. lanuginosus* (PDB ID: 1EIN) (Brzozowski et al., 2000).

### Molecular Docking Analysis of PEXANL1

**FIGURE 5:**
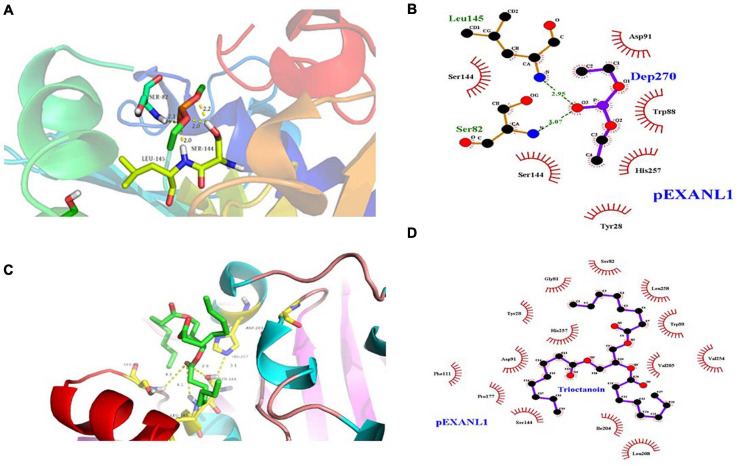
Molecular docking analysis of PEXANL1 with its ligands. **(A)** Active site showing DEP ligand orientation, **(B)** 2D schematic presentation of DEP interaction with active site residues, **(C)** Active site showing tricaprylin ligand orientation, and **(D)** 2D schematic presentation of tricaprylin orientation at active site residues.

Lipase and serine proteases are highly similar, and lipase’ activity is inhibited by hydroxymethyl diethyl phosphate (DEP) (Derewenda et al., 1992). In this study, we analyzed the PEXANL1 and DEP ligand interaction using molecular docking. DEP showed a strong binding affinity for PEXANL1 (binding energy was −6.89 kcal/mol). Based on the DEP and PEXANL1 complex structure, DEP was localized in the active center of the complex. Besides, it formed a hydrogen bond with the H on the carbonyl oxygen present on the side chain of the active site (Ser 144) of PEXANL1 (Figure 5A), which is in line with the previous report (Rajulapati et al., 2018). The docked complexes showed that DEP formed hydrophobic bonds with Ser144, Ser145, Tyr28, His257, Trp88, and Asp91 of PEXANL1 (Figure 5B). DEP was firmly bound to the active center of the complex by forming strong hydrogen bonds with the oxygen anion holes in Ser82 and Leu145 of PEXANL1 (Figure 5B).

Lipase hydrolyzes medium and long-chain fatty acid glycerides efficiently. Certain lipases hydrolyze glycerol chains selectively based upon their position. In this study, we used the molecular docking method to study PEXANL1 and tricaprylin ligand interaction. The tricaprylin showed a stronger binding affinity to PEXANL1 (binding energy was −8.74 kcal/mol) than the DEP ligand. Figure 5C depicts the tricaprylin docking on the PEXANL1. The PEXANL1 was positioned through hydrogen bonds in the active rift (Ser82, Leu145, and Ser 144) and hydrophobic interactions (Gly81, Tyr28, Phe111, Pro177, Asp 91, Ser144, Ile204, Leu208, Val205, Leu258, Trp88, and Val254) (Figure 5D). Aromatic amino acid residues involved in the formation of catalytic cracks promote catalysis. The amino acid residues, Ser82 and Leu145, formed hydrogen bonds with the carbonyl oxygen of the ligands. These residues recognize and regulate the active site of the ligand.

### Low-Resolution Structure Analysis of PEXANL1 by Small-Angle X-Ray Scattering (SAXS)

**FIGURE 6:**
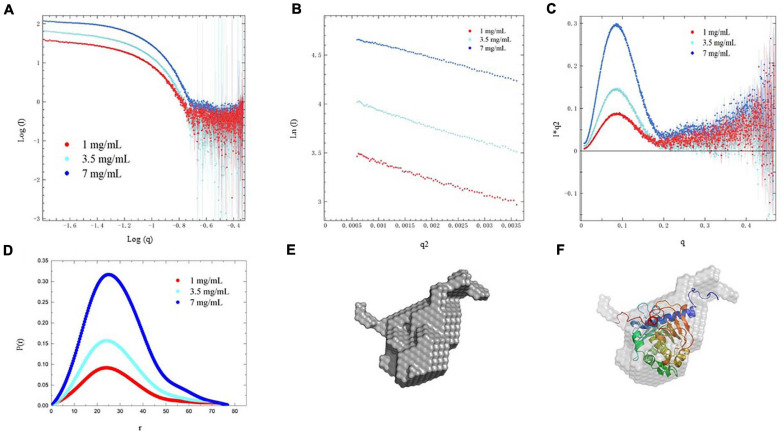
SAXS analysis of PEXANL1. **(A)** SAXS intensity profile of PEXANL1, **(B)** Guinier plot of the SAXS intensities, **(C)** Kratky plot analysis of PEXANL1, **(D)** *P*(*r*) curve of PEXANL1 as a function of *r*, **(E)** *ab initio* shape of PEXANL1, **(F)** superposition of *ab initio* model with homology modeled PEXANL1.

SAXS data analysis was employed to investigate the solution conformation of PEXANL1. Scattering data of PEXANL1 concentration (1, 3.5, and 7 mg/ml) was collected to conduct SAXS analysis. The PEXANL1 structural parameters were acquired using SAXS data processing, and the outcomes of this analysis are summarized in Supplementary Table 4. The uniformity of scattering intensity curves of PEXANL1 at different concentrations validated the absence of aggregation and inter-particle correlation effects (Figure 6A). PEXANL1 Guinier plot revealed that PEXANL1’s radius of gyration (Rg) was 2.00 ± 0.50 to 2.24 ± 0.21 nm (at PEXANL1 concentrations of 1, 3.5, and 7 mg/mL), and PEXANL1’s linearity confirmed its monodisperse state in the solution (Figure 6B). The molecular weight of PEXANL1 was found to be 31.62 kDa using SAXS data and SAXSMoW tool, which is in line with the theoretical molecular weight, as per the amino acid sequence analysis (31.5 kDa) (Supplementary Table 4). The Kratky plot analysis of the PEXANL1 showed that it was completely folded in solution (Figure 6C). It showed a major symmetrical peak, *q* < 2 nm^–1^, which did not decay to near zero at higher *q*-values and maintained a slight elevation. Furthermore, Kratky analysis showed that there was some flexibility due to the overexpression of N-terminus and His tag C-terminus PEXANL1 amino-acids. The indirect Fourier transform evaluated the distance distribution function [*P*(*r*)] over a range of 0.17–3.55 nm^–1^. It showed that Dmax and Rg values were 7.8 and 2.1 nm, respectively (Figure 6D). Multiple independent cycles of *ab initio* modeling using DAMMIF were computed without symmetry restrictions, and the average was calculated using DAMAVER to produce a low-resolution *ab initio* shape for PEXANL1 (Figure 6E). The *ab initio* derived pseudo-atom model was compact and revealed a single catalytic domain with two tails in the form of a hexahistidine tag at C-terminal and overexpressed amino acids at N-terminal. The pseudo-atom model derived from the *ab initio* analysis validated the structure of the comparatively modeled PEXANL1 model (Figure 6F).

## Conclusion

In this study, we characterized an sn-1, 3 lipases from *A. niger*, which was designated as PEXANL1. PEXANL1 was heterologously expressed, purified, and biochemically characterized. The highest lipase activity of 66.5 ± 1.4 U/mL was attained by using 1% methanol and 96 h induction time. The enzymatic properties of PEXANL1 were also investigated. The optimum pH and temperature for PEXANL1 were 4.0 and 40°C, respectively, different from these of recombinant enzymes and natural enzymes expressed in *E. coli*, due to different expression strategies and glycosylation modifications. Besides, we observed that PEXANL1 could maintain its activity in a wide pH range (2–8), and it was extensively tolerant to multiple organic solvents. Triton X-100, Tween 20, Tween 80, and SDS (0.01% w/v) enhanced the enzymatic activity of PEXANL1. Effects of different metal ions on PEXANL1 activity was also studied. All tested metal ions inhibited the PEXANL1 activity. With the aid of ultrasound, the time for PEXANL1 to catalyze the esterification of glycerol and oleic acid to synthesize 1,3-diglycerides was significantly shortened. The predicted 3D structure of PEXANL1 using comparative modeling was stable and compact. The docking analysis validated that Ser162, His274, and Asp217 residues were involved in the catalytic function of PEXANL1. Small-angle X-ray scattering analysis demonstrated the monomer properties of PEXANL1 in solution. The *ab initio* model of PEXANL1 superposed well with its predicted 3D structure. This work lays the foundation for the industrial application of recombinant lipase and provides a basis for the subsequent study on the rational design of suitable crystal mutants of *A. niger* lipase.

## Data Availability Statement

The datasets generated for this study can be found in online repositories. The names of the repository/repositories and accession number(s) can be found below: https://www.ncbi.nlm.nih.gov/, MK592411.1.

## Author Contributions

SX: conceptualization, methodology, investigation, visualization, and writing-original draft. RZ: biochemical characterization of PEXANL1. KC: homology modeling and structure validation of PEXANL1. YC: molecular docking analysis. YH: SAXS data analysis. CL, XZ, and QZ: writing-review and editing. LH: funding acquisition, project administration, supervision, and writing-review and editing. All authors contributed to the article and approved the submitted version.

## Conflict of Interest

The authors declare that the research was conducted in the absence of any commercial or financial relationships that could be construed as a potential conflict of interest.

## Footnotes

1 https://web.expasy.org/translate/

2 http://bioinf.cs.ucl.ac.uk/psipred/

3 http://2struc.cryst.bbk.ac.uk/twostruc

4 https://spdbv.vital-it.ch/

5 http://services.mbi.ucla.edu/SAVES/

6 https://prosa.services.came.sbg.ac.at/prosa.php

7 http://www.PyMOL.org

8 http://pubchem.ncbi.nlm.nih.gov

9 http://www.PyMOL.org

10 https://web.expasy.org/protparam/

11 http://www.cbs.dtu.dk/services/NetNGlyc/
