# Supplementary material for: Gene Expression, Biochemical Characterization of a sn-1, 3 Extracellular Lipase From Aspergillus niger GZUF36 and Its Model-Structure Analysis
Source: Front Microbiol. 2021 Mar 12;12:633489. doi: 10.3389/fmicb.2021.633489 (PMC7994357; doi:10.3389/fmicb.2021.633489)
Supplement: Supplementary file 1 [file Data_Sheet_1.docx]

**Supplementary material**

**Gene expression, biochemical characterization of a sn-1, 3 extracellular lipase from *Aspergillus niger* GZUF36 and its model-structure** **analysis**

Shuqi Xing^1, 2^, Ruonan Zhu^1, 2^, Kai Cheng^1, 2^, Yangyang Cai^1, 2^, Yuedan Hu^1, 2^, Cuiqin Li^1, 2, 3,4^, Xuefeng Zeng^1, 2, 4^, Qiujin Zhu^1, 2, 4^, Laping He^1, 2, 4,^ *

^1^ Key Laboratory of Agricultural and Animal Products Store & Processing of Guizhou Province, Guizhou University, Guiyang 550025, PR China

^2^ College of Liquor and Food Engineering, Guizhou University, Guiyang 550025, PR China

^3^ School of Chemistry and Chemical Engineering, Guizhou University, Guiyang 550025, PR China

^4^ Key Lab of Fermentation Engineering and Biopharmacy, Guizhou University, Guiyang 550025, PR China

****Correspondence***:

Laping He

helaping@163.com

**
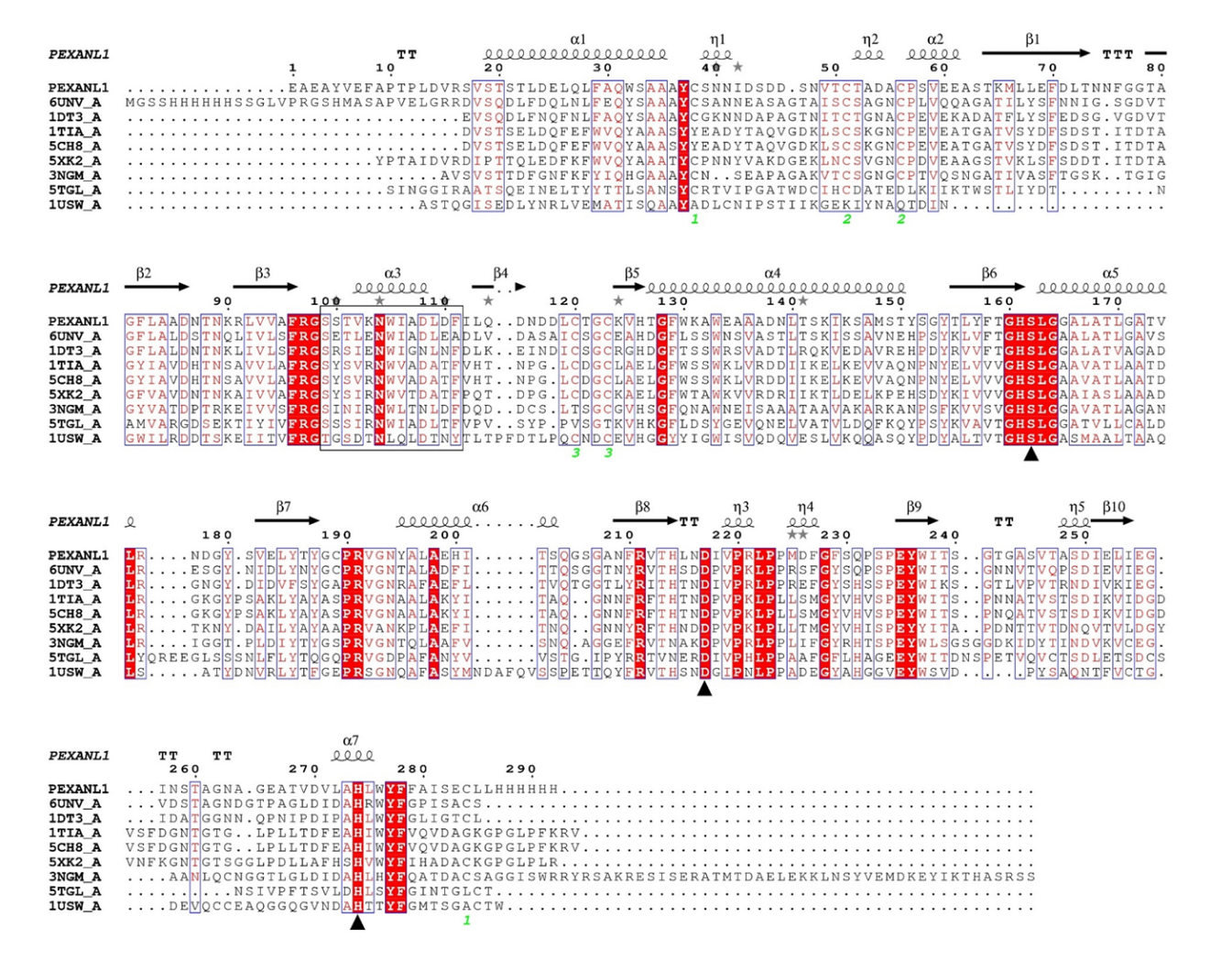
**

**FIGURE S1** The multiple sequence alignment (MSA) of PEXANL1 showing the conserved (red background), semi-conserved (red colour letters) and dispersive amino acids (black colour letters). The lipases indicated by the pdb access number was as follows: *Rasamsonia emersonii* lipase (PDB ID: 6UNV), *Thermomyces lanuginosus* lipase (PDB ID: 1DT3), *Penicillium camemberti* lipase (PDB ID: 1TIA), *Penicillium cyclopium* (PDB ID: 5CH8), *Aspergillus oryzae* lipase (PDB ID: 5XK2), *Fusarium graminearum* lipase (PDB ID: 3NGM) and the ferulic acid esterase from *Aspergillus niger* (PDB ID: 1USW). The deduced catalytic triad is identified as a triangle. The secondary structure based on the three-dimensional structure of the prediction model of PEXANL1 was displayed by ESPript 3 softwares. The amino acid sequence corresponding to the lid domain was represented by a square box located behind the FRG conserved motif.The green number pairs were inferred disulfide bonds.


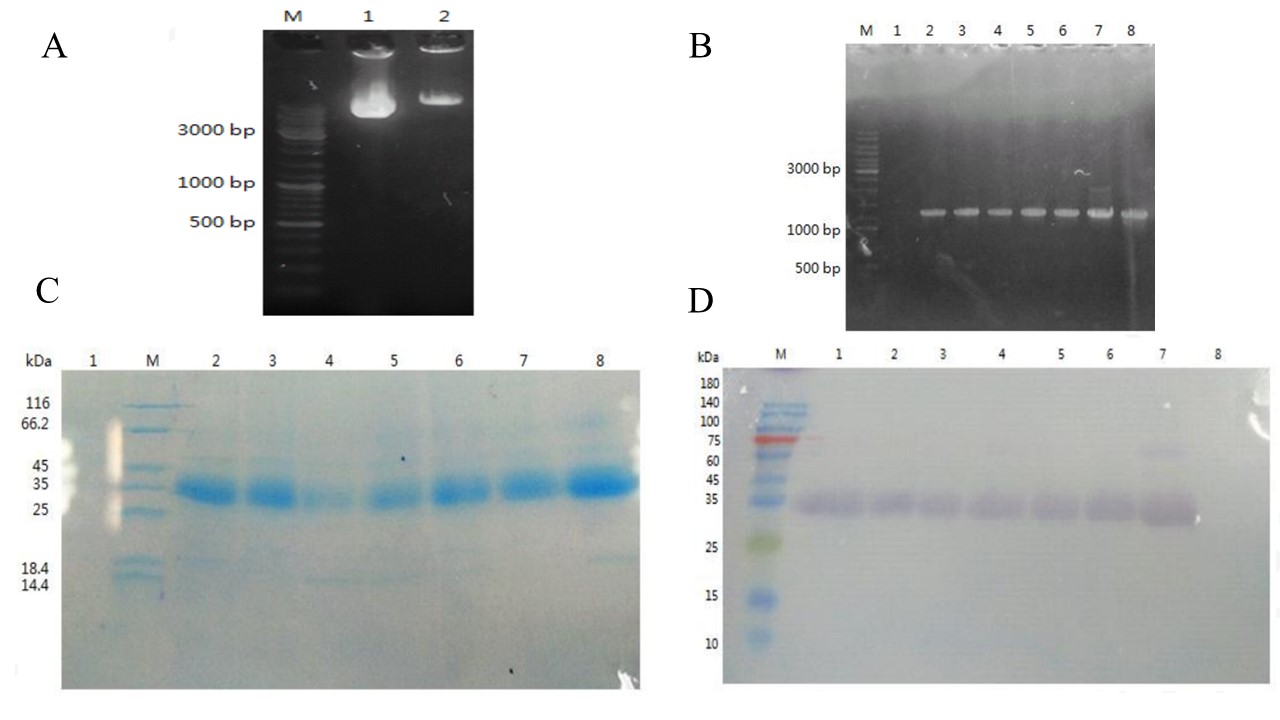


**FIGURE S2** Cloning and expression of *Aspergillus niger* GZUF36 lipase in *Pichia pastoris* (PEXANL1). (A) Linear electrophoresis analysis of recombinant plasmids. Lane M: the molecular weight marker. Lane 1: Linearized plasmid; Lane 2: Unlinearized plasmid. (B) Colony PCR analysis of transformants. Lane M: the molecular weight marker. Lane 1: The negative strain; Lane 2-8: The positive strain A, B, C, D, E, F and G. (C) SDS-PAGE analysis of fermentation supernatant of transformants. Lane M: the molecular weight marker. Lane 1: The negative strain; Lane 2-8: The culture supernatant of PEXANL1 corresponds to these positive strains A, B, C, D, E, F and G. (D) WB analysis of fermentation supernatant of transformants. Lane M: the molecular weight marker. Lane 1-7: The culture supernatant of PEXANL1 corresponds to these positive strains A, B, C, D, E, F and G.; Lane 8: The negative strain.

**
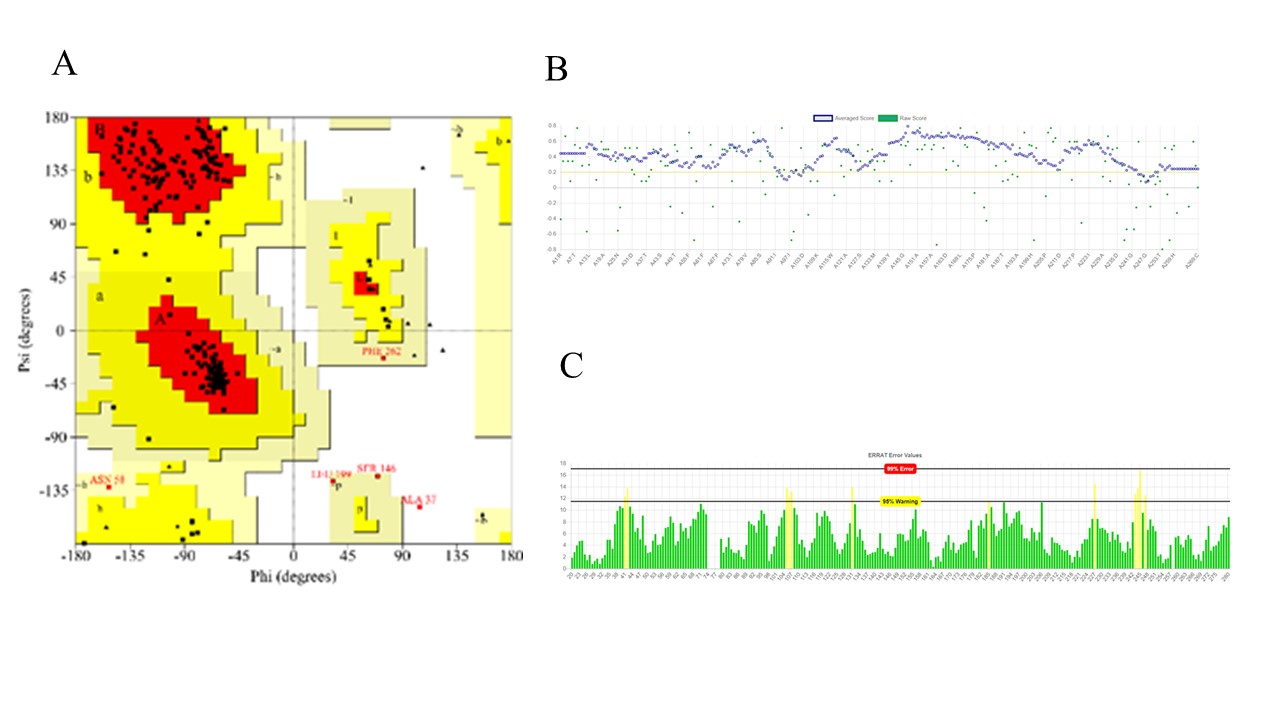
**

**FIGURE S3** 3-D structure model evaluation of PEXANL1. (A) Ramachandran plot (UCLA SAVES software) showing the most favorable, additional allowed and generously allowed regions of amino acid residues. (B) VERIFY 3D showed the score was more than 0.2. (C) Errat plot analysis showing the quality of the PEXANL1 modeled structure.

**TABLE S1** Gradient elution conditions for analyzing the composition of glycerides. The organic solvent used was chromatographic grade, and the water was ultrapure water.

| Time (min) | acetonitrile(%) | water(%) | n-hexane/isopropanol (4:5, v/v) (%) |
| --- | --- | --- | --- |
| 0 | 70 | 30 | 0 |
| 10 | 100 | 0 | 0 |
| 12 | 0 | 50 | 50 |
| 22 | 0 | 50 | 50 |
| 23 | 100 | 0 | 0 |
| 24 | 70 | 30 | 0 |
| 28 | 70 | 30 | 0 |

**TABLE S2** Purification parameter of PEXANL1.

| Purification steps | Total activity (U)^a^ | Total  protein  (mg)^b^ | Specific  activity  (U/mg) | Fold purification | Yield (%) |
| --- | --- | --- | --- | --- | --- |
| Fermentation supernatant | 2604.9 | 182.16 | 14.3 | 1 | 100 |
| ultrafiltration concentrate | 2404.3 | 149.19 | 16.1 | 1.12 | 92.3 |
| Ni-NTA | 1670.9 | 8.08 | 206.8 | 12.8 | 64.1 |

Notes: ^a^ One unit (U) corresponds to one micromole of fatty acid released per minute using olive oil as substrate under the experimental conditions used. Activity measurements are described in Materials and methods 2.4. ^b^The protein concentration was determined using the Pierce BCA protein assay kit, and bovine serum albumin was used as the standard.

**TABLE S3** Comparative analysis of the enzymatic properties of PEXANL1, lipase expressed in *E. coli* and native lipase.

| Source | Optimal pH | pH stability | Optimum temperature (°C) | temperature stability  (°C) |
| --- | --- | --- | --- | --- |
| lipase expressed in *E. coli* | 4 | 2-8 | 35 | <50 |
| native lipase | 6.5 | 6-7 | 35 | 50 |
| PEXANL1 | 4 | 2-8 | 40 | <50 |

**TABLE S4 SAXS scattering collection parameters and derived parameters of PEXANL1**

| Data-collection parameters | PEXANL1 | | |
| --- | --- | --- | --- |
| Instrument | BL19U2 | | |
| Wavelength (nm) | 0.09184  0.0789-4.6838  1.5 | | |
| Q range （nm^-1^） |  |  |  |
| Exposure time（s） |  |  |  |
| Protein Concentration（mg/mL） | 1 | 3.5 | 7 |
| Temperature（℃） | 25 | 25 | 25 |
| Structural parameter |  |  |  |
| Q range（nm^-1^）used for Rg analysis | 0.17-3.55 | 0.17-3.55 | 0.17-3.55 |
| I(0) au from Guinier | 17.26±0.042 | 16.90±0.018 | 16.40±0.023 |
| Rg from Guinier（nm） | 2.24±0.21 | 2.15±0.07 | 2.00±0.50 |
| I(0) au from P(R) | 17.30 | 16.90 | 16.70 |
| Rg from P(R)（nm） | 2.30 | 2.16 | 2.10 |
| Dmax（nm） | 8.1 | 8.0 | 7.8 |
| Porod volume estimate (nm^3^) | 39.21 | 43.75 | 39.98 |
| Molecular Mass (MM) determination |  |  |  |
| MM calculated from I(0) (kDa)（kDa） | 33.09 | 34.93 | 31.47 |
| Theoretical mass MM (kDa)（kDa） | 31.5 | 31.5 | 31.5 |
| Software employed |  |  |  |
| Data processing | PRIMUS | PRIMUS | PRIMUS |
| P(R) function calculation | GNOM | GNOM | GNOM |
| Ab initio modeling | DAMMIF | DAMMIF | DAMMIF |
| Validation and averaging | DAMAVER | DAMAVER | DAMAVER |
| 3-D graphical representation | Pymol | Pymol | Pymol |
| Modeling parameters |  |  |  |
| Chi^2^ | 0.017 | 0.015 | 0.018 |
| NSD | 0.022 | 0.022 | 0.021 |
